# QUEST Perspective Study (QPS) to measure the understanding by patients and healthcare professionals of surgical breast reconstruction clinical trials: QUEST Trials A & B

**DOI:** 10.1186/1745-6215-12-S1-A105

**Published:** 2011-12-13

**Authors:** Zoe E Winters, Judith Mills, Marie Emson, Judith M Bliss, Rob Horne

**Affiliations:** 1 University of Bristol, School of Clinical Sciences, Bristol, UK; 2 Clinical Trials & Statistic Unit (CTSU-ICR), The Institute of Cancer Research, Sutton, Surrey, UK; 3 School of Pharmacy, London University, UK

## Introduction

The QUEST trials are the first surgical trials comparing different types (A) and timing (B) of Latissimus dorsi (LD) reconstruction with a primary outcome of quality of life (QoL) in an attempt to improve clinical evidence. Surgical trials are challenging, necessitating a feasibility study, and the QUEST Perspectives Study (QPS), to assess the acceptability of randomisation from the perspectives of patients and healthcare professionals (HCPs).

## Methods

To maximise information from both patients and HCPs who either accept or decline to participate in the trial, it was agreed that a mixed methodologies should be used in QPS. The questionnaires ‘Patient views on QUEST (PVQ) would be given to patients after they have or have not consented /randomised to QUEST. Twenty acceptors will be purposively selected for telephone interview 1 month post-operatively. The HCP questionnaire called PEERS (PERCEPTIONS OF EQUIPOISE EVIDENCE and RANDOMISATION SURVEY) will be requested from all PIs approached to participate in QUEST.

## Results

QPS for patients consists of an ‘acceptors’, and a ‘decliners’ questionnaire regardless of trial participation. Both questionnaires have a free ‘writing task’ where patients can record any views they might have about QUEST. By comparison, the decliners have formulated questions to respond to on a number of statements about randomisation, their surgeon and their surgical preferences. PEERS comprises a number of statements on which the HCPs are asked to rate their agreement on their views regarding clinical equipoise and the randomisation design in QUEST.

## Conclusion

By integrating the QPS, we will gain a far better insight into why patients wish to enter surgical randomised clinical trials and so facilitate the design of future trials in breast reconstruction. Furthermore, capturing HCPs views are essential in this trial because they need to relay the meaning of clinical equipoise to patients based on the current evidence.

